# Association Between Frailty and Pelvic Organ Prolapse in Elderly Women: A Retrospective Study

**DOI:** 10.1007/s00192-024-05898-x

**Published:** 2024-08-26

**Authors:** Daisuke Obinata, Makoto Hara, Sho Hashimoto, Ken Nakahara, Tsuyoshi Yoshizawa, Junichi Mochida, Kenya Yamaguchi, Satoru Takahashi

**Affiliations:** 1 Department of Urology, Nihon University School of Medicine, Tokyo, Japan; 2 Division of Neurology, Department of Medicine, Nihon University School of Medicine, Tokyo, Japan

**Keywords:** Ageing, Pelvic organ prolapse, Elderly people, Frailty, Sarcopenia

## Abstract

**Introduction and Hypothesis:**

This study evaluated the association between pelvic organ prolapse (POP), frailty, and sarcopenia to explore how POP treatment can extend healthy life expectancy in elderly women.

**Methods:**

We conducted a retrospective study of prospectively collected data, comparing women with mild POP (stages 0–II) with those with advanced POP (stages III and IV). The inclusion criteria for this study were women who visited the clinic with at least one symptom of pelvic floor dysfunction and underwent imaging studies between April 2020 and November 2022. Initially, 119 patients met these inclusion criteria. Patients were excluded if they had a history of previous POP treatment, did not respond to the study survey, or were lost to follow-up. After applying these exclusion criteria, 82 patients were included in the final analysis, of whom 65 underwent surgery (laparoscopic sacrocolpopexy, colpocleisis, tension-free vaginal tape, and native tissue repair). Assessments included POP Quantification, Kihon Checklist, Pelvic Organ Prolapse Quality of Life (P-QOL) questionnaire, International Prostate Symptom Score (IPSS), Overactive Bladder Symptom Score (OABSS), and Incontinence Symptom Questionnaire (ICIQ-SF). Pelvic muscles were measured using MRI or CT. Immunohistochemical analysis of estrogen receptor alpha (ERα), estrogen receptor beta , and androgen receptor was performed on surgical specimens from 43 patients.

**Results:**

The median age of participants was 75 years. Of the 82 patients, 48 (58.5%) were classified as frail or pre-frail, and 22 (26.8%) exhibited motor impairment. Advanced POP (stages 3 and 4) was seen in 41 patients. These patients had more motor function impairments (advanced, 16; mild, 6; *p* = 0.01). Patients with advanced POP had poorer P-QOL, ICIQ-SF (median: 9.5 vs 4, *p* = 0.006) and OABSS (7 vs 4, *p* = 0.008) scores, and smaller pubococcygeus muscle diameter (2.5 vs 3 cm, *p* = 0.017). Postoperatively, significant improvements were seen in P-QOL (all domains except personal relationships: *p* < 0.001), total IPSS (11 vs 4, *p* < 0.001), OABSS (6 vs 5, *p* = 0.033), and ICIQ-SF scores (6 vs 2, *p* < 0.001). ERα expression was associated with preoperative frailty (*r* = −0.37, *p* = 0.014).

**Conclusions:**

Advanced POP correlates with poorer QOL, worse urinary symptoms, and reduced pubococcygeus muscle diameter, consistent with sarcopenia, compared with mild POP.

**Supplementary Information:**

The online version contains supplementary material available at 10.1007/s00192-024-05898-x.

## Introduction

The World Health Organization (WHO) has emphasized the importance of healthy life expectancy for several decades [1, 2]. This metric represents the average life expectancy minus the time spent on declining activities of daily living and quality of life (QOL). The discrepancy between average life expectancy and healthy life expectancy reveals the duration of care and support that is required to ensure longevity. As life expectancy has increased in the twentieth century [3], it is crucial to assess the number of years an elderly individual can anticipate living in good health, considering age-specific mortality rates, the prevalence of illness, and the incidence of disability. The average life expectancy in Japan increased between 1990 and 2013, with figures increasing from 76.0 to 80.1 years for men and from 82.0 to 86.4 years for women. Similarly, healthy life expectancy also showed improvement; men experienced an increase from 68.1 to 71.1 years, whereas women saw an increase from 72.2 to 75.6 years over the same period [4]. Despite these gains, the gap between the average life expectancy and healthy life expectancy persistently remains over 10 years [4], highlighting the urgent need to extend healthy life expectancy.

The influence of frailty on healthy life expectancy is significant. Frailty is a multisystem disorder that renders individuals susceptible to stressors and increases the likelihood of adverse health outcomes [5]. Frailty, characterized by a decline in functional capacity, can be attributed to various factors, including skeletal muscle mass and function. Sarcopenia, defined as the loss of muscle mass, is associated with frailty in musculoskeletal aging. A study conducted on healthy life expectancy and frailty in Europe between 2004 and 2015 observed that 70-year-old women spent approximately 25% of their remaining life expectancy in a frail state [6]. Additionally, mortality rates have been found to increase among frail men and women [7].

Pelvic organ prolapse (POP) is a prevalent condition in elderly women and is characterized by symptomatic descent of the uterus, bladder, and bowel from their normal anatomical positions, resulting in lower urinary tract dysfunction and a reduced QOL [8–10]. POP affects ≥ 40% of elderly women, primarily because of childbirth and aging. This condition can be improved by pelvic floor muscle training [11].

The weakening or impairment of the pelvic floor muscles is a key factor in the development of POP [12]. Pelvic floor muscle weakness leads to insufficient support for the pelvic organs, causing them to descend, resulting in symptoms associated with POP. This relationship between POP and pelvic floor muscle weakness highlights the importance of interventions aimed at strengthening these muscles to manage and potentially reverse POP. Understanding the extent of muscle impairment in patients with POP is crucial for developing effective treatment strategies. Therefore, age-related loss of muscle mass and aging of the pelvic floor muscles may be closely related to frailty and sarcopenia. However, the specific details of these relationships are not yet fully understood. Evaluating the relationship between frailty, sarcopenia, and POP is important, as it may affect healthy life expectancy in older women. This could also lead to better surgical outcomes and postoperative recovery for those undergoing treatment for POP.

In this study, we aimed to evaluate the association between POP, frailty, and sarcopenia in elderly women. Specifically, we compared the outcomes of patients with advanced POP (stages III and IV) with those with mild or no POP (stages 0 to II). Our objective was to determine how POP correlates with frailty and sarcopenia, and to understand the importance of addressing these conditions in the treatment of POP. Additionally, we performed immunostaining for estrogen receptor (ER) to investigate its relationship with frailty and sarcopenia. Estrogen and ER play a crucial role in skeletal muscle homeostasis and exercise capacity [13]. In postmenopausal women, the decline in ER levels leads to decreased muscle mass and strength, contributing to conditions such as sarcopenia and frailty [13]. By assessing ER expression, in addition to other sex steroid hormone receptors, we aimed to gain further insight into the biological mechanisms underlying these conditions and their impact on POP.

## Materials and Methods

**Fig. 1:**
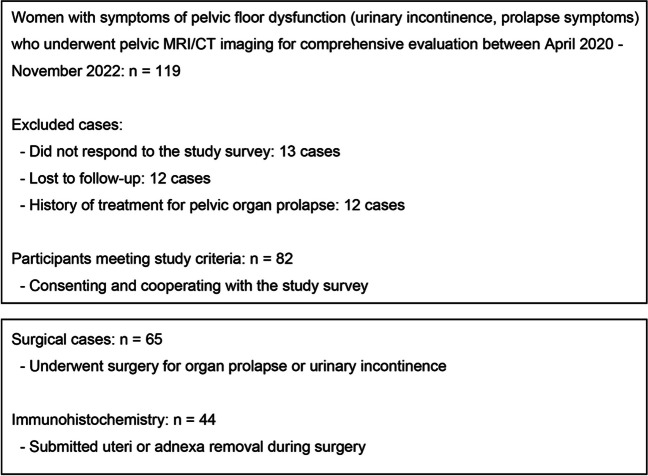
Flowchart of patient selection and inclusion in the study

This retrospective cohort study of prospectively collected data compared women with mild POP (stages 0–II) with those with advanced POP (stages III and IV) to determine if advanced prolapse is more commonly associated with frailty and sarcopenia. The study was conducted with the approval of our Institutional Review Board (IRB). All participants provided informed consent prior to inclusion in the study. Details regarding IRB approval and ethical considerations are provided in the Statements and Declarations section. The inclusion criteria for the study were women who visited the clinic with complaints of at least one symptom of pelvic floor dysfunction, such as urinary incontinence, or prolapse symptoms, and underwent imaging studies between April 2020 and November 2022. At our facility, we perform imaging studies such as pelvic CT or MRI for new patients presenting with symptoms of pelvic floor dysfunction to further evaluate other potential diseases and the pelvic structure. These imaging studies were conducted with the patient’s consent. It should be noted that this approach is not a standard practice for urogynecological investigations, where pelvic ultrasound is typically the preferred modality. Only patients who provided informed consent and met the study survey requirements were included. The exclusion criteria included patients who did not respond to the study survey, those lost to follow-up, and those with a history of previous POP treatment. Of the 119 patients initially approached, 13 did not respond to the study survey, 12 were lost to follow-up, and 12 had a history of previous POP treatment. Therefore, 82 patients met the study criteria, provided written informed consent, and were recruited for the study, resulting in a response rate of 68.9% (Fig. 1).

The POP Quantification (POP-Q) [14] evaluations were performed in an outpatient setting, and the data were recorded in clinical files. POP was primarily assessed through clinical pelvic examinations using the POP-Q system. At our facility, we routinely performed imaging studies such as pelvic CT or MRI for new patients presenting with symptoms of pelvic floor dysfunction for comprehensive evaluation. The Kihon Checklist was used to assess frailty [15], with scores recorded in the clinical files. The Kihon Checklist is widely used in Japan to identify elderly individuals at risk of needing support or care. It consists of 25 items covering multiple domains, including physical function, nutrition, socialization, and cognitive function. Each item is scored, and the total score categorizes individuals into nonfrail, pre-frail, or frail. Scores of 4–7 indicate pre-frailty, whereas scores of 8 or higher indicate frailty. A study has shown that the total Kihon Checklist score correlates closely with the number of frailty phenotypes defined by the Cardiovascular Health Study criteria [16]. We evaluated declines in each domain and identified individuals as pre-frail with scores of 4–7 and frail with scores of ≥ 8. The impact of POP on QOL was assessed using the Pelvic Organ Prolapse Quality of Life (P-QOL) questionnaire [17, 18], with responses documented in clinical files. The International Prostate Symptom Score (IPSS) [19], Overactive Bladder Symptom Score (OABSS) [20], and Incontinence Symptom Questionnaire (ICIQ-SF) [21] were used to assess urinary symptoms, with all results documented in clinical files. Follow-up interviews were conducted in January 2023 using a retrospective mail survey and responses were added to the clinical files.

Magnetic resonance imaging or CT scans were employed to measure the maximum diameters of the internal obturator internus and gluteus maximus, as well as the pubococcygeus muscles. These diameters were measured three times, and the mean values were calculated and recorded in the clinical files. Although there are no universally established standards or cutoff values specifically for these muscle measurements in the context of sarcopenia or POP, our measurements were based on previously published methods [22].

Of the 82 patients included in the study, 65 underwent surgery for POP or stress urinary incontinence. Postoperative POP-Q stages were assessed during follow-up visits in the outpatient clinic. Among the surgical cohort, 43 patients underwent intraoperative removal of uterine and ovarian specimens owing to pathology, and these residual specimens were used retrospectively to evaluate estrogen receptor alpha (ERα) and beta (ERβ) and androgen receptor (AR) expression with an immunoreactivity (IR) score [23]. Histological assessment of the extracted pathological specimens was planned at the time of the study design. Immunohistochemical analysis was conducted using the streptavidin–biotin amplification method, as previously described [23, 24]. Briefly, monoclonal antibodies against ERα (1:100 dilution, SC8002; Santa Cruz Biotechnology, CA, USA), ERβ (1:100 dilution, SC390243, Santa Cruz Biotechnology), and a polyclonal antibody against AR (1:3,200 dilution, A9;53, Sigma, St Louis, MO, USA) were applied overnight to the samples. These antibodies were used in accordance with previous reports [25]. The secondary antibody used was Histofine Simple Stain MAX-PO (Nichirei, Tokyo Japan). Antigen–antibody complexes were visualized using 3,30-diaminobenzidine (DAB) solution (1 mM DAB, 50 mM Tris–HCl buffer [pH 7.6], and 0.006% H2O2). The specificity of these antibodies has been previously confirmed. The IR score (0–8 points) was calculated as the sum of the proportion and intensity of immunoreactivity, with the following definitions: proportion (0, none; 1, < 1/100; 2, 1/100 to 1/10; 3, 1/10 to 1/3; 4, 1/3 to 2/3; and 5, > 2/3); intensity (0, none; 1, weak; 2, moderate; and 3, strong). The IR score is a widely used method in immunohistochemistry to quantify the expression levels of specific receptors in tissue samples. This method combines both the proportion of positive cells and the intensity of staining to provide a comprehensive assessment of protein expression in tissues. This approach helps to evaluate whether the decline in ER levels affects muscle mass, as previous studies have shown [13].

The number of cases in this study was calculated using G*Power software [26] with an effect size of 0.4, power of 0.8, and significance level of 0.05. The Mann–Whitney *U* test was used for between-group comparisons with continuous variables. For comparisons of ordinal and nominal variables, the Chi-squared test was employed. Preoperative and postoperative comparisons were performed using the Wilcoxon matched-pair signed-rank test. A *p* value of < 0.05 was considered statistically significant for all tests.

## Results

All 82 participants were assessed. The median (interquartile range) of the participants was 75 (72–79) years. Among them, 48 (58.5%) were classified as frail or pre-frail and 22 (26.8%) exhibited motor impairment. Five patients (6.1%) were classified as stage 0 POP-Q, 2 (2.4%) as stage 1, 34 (41.5%) as stage 2, 32 (39.0%) as stage 3, and 9 (11.0%) as stage 4. In this study, stages 3 and 4 were defined as advanced POP (*n* = 41, 50.0%). Patients with advanced POP were slightly older on median with interquartile range compared with those with mild POP, but the difference was not significant (median age: 76.0 [72.5–80.5] vs 75.0 [71.5–78.0], *p* = 0.2; Table 1). According to the Kihon Checklist, there was a higher incidence of motor function impairment in patients with advanced POP-Q than in those with a mild POP-Q stage (16 vs 6, *p* = 0.01; Table 2). Based on this analysis, although pre-frail and frail conditions were more common among patients with advanced POP, the difference was not statistically significant, with a *p* value just above the threshold (Table 2, *p* = 0.058). However, when focusing exclusively on surgically treated cases, these conditions showed a statistically significant increase in the prevalence in patients with advanced POP (24 vs 10, *p* = 0.018; Supplementary Table 1). In the P-QOL assessment, patients with advanced POP had significantly poorer outcomes in all domains, except for personal relationships, than those with mild POP (general health perception: median 50 vs 25, *p* = 0.002; prolapse impact: median 100 vs 33.3, *p* < 0.001; role limitations: median 50 vs 16.6, *p* < 0.001; physical limitations: median 66.6 vs 33.3, *p* < 0.001; social limitations: median 33.3 vs 0, *p* = 0.002; emotions: median 55.5 vs 11.1, *p* < 0.001; sleep/energy: median 33.3 vs 16.6, *p* = 0.007; severity measures: median 50.0 vs 25.0, *p* = 0.011; Table 1). In contrast, the IPSS showed no significant association between POP severity and voiding symptoms (Q1, 3, 5, and 6 total), storage symptoms (Q2, 4, and 7 total), total score, or QOL (Table 1). Conversely, the ICIQ-SF and OABSS indicated significantly worse outcomes in patients with more advanced POP (ICIQ-SF: median 9.5 vs 4, *p* = 0.006; OABSS: median 7 vs 4, *p* = 0.008; Table 1). In the pelvic floor muscle assessment, pubococcygeus muscle diameter was significantly shorter in patients with advanced POP than in those with mild POP (median 2.5 cm vs 3 cm, *p* = 0.017; Table 1). No significant differences were observed for the other muscles.

**Table 1:** Comparison between mild and advanced POP cases (*n* = 82)

	POP-Q		
Median (IQR)	Mild stage ≤ II, *n* = 41	Advanced stage ≥ III, *n* = 41	*p* value
Age (years)	75.0 (71.5–78.0)	76.0 (72.5–80.5)	0.2
P-QOL			
General health perception	25 (25–50)	50 (25–75)	0.002
Prolapse impact	33.3 (0–66.6)	100 (66.6–100)	< 0.001
Role limitations	16.6 (0–33.3)	50 (33.3–83.3)	< 0.001
Physical limitations	33.3 (0–50.0)	66.6 (33.3–100)	< 0.001
Social limitations	0 (0–22.2)	33.3 (0–55.5)	0.002
Personal relationships	0 (0)	0 (0)	0.04
Emotions	11.1 (0–33.3)	55.5 (22.2–77.7)	< 0.001
Sleep/energy	16.6 (0–33.3)	33.3 (16.6–66.6)	0.007
Severity measures	25.0 (8.3–50.0)	50.0 (25.0–66.6)	0.011
LUTS scores			
IPSS—sum of voiding domains	4 (2–8)	5 (2–7)	0.6
IPSS—sum of storage domains	4 (2–8)	6 (4–8)	0.11
IPSS total	9 (4–14)	12 (8–15)	0.2
IPSS QOL	4 (3–5)	5 (3–6)	0.14
ICIQ-SF	4 (0–8)	9.5 (4–14.5)	0.006
OABSS	4 (2–6)	7 (4–10.5)	0.008
Pelvic floor muscle diameter			
Obturator internus	12.7 (11.2–14.7)	12.0 (8.9–13.8)	0.18
Gluteus maximus	21.4 (19.1–23.6)	21.5 (17.8–24.5)	0.8
Pubococcygeus	3.0 (2.5–3.5)	2.5 (1.7–3.2)	0.017

*IQR* interquartile range, *P-QOL* Pelvic Organ Prolapse Quality of Life, *LUTS* lower urinary tract symptoms, *IPSS* International Prostate Symptom Score, *QOL* quality of life, *ICIQ-SF* Incontinence Symptom Questionnaire, *OABSS* Overactive Bladder Symptom Score, *POP-Q* Pelvic Organ Prolapse Quantification

**Table 2:** Comparison of the Kihon Checklist results in mild and advanced cases (*n* = 82)

	POP-Q		
Number of positive cases	Mild stage ≤ II, *n* = 41	Advanced stage ≥ III, *n* = 41	*p* value
Decline in motor function	6	16	0.01
Malnutrition	0	0	–
Decline in oral function	5	10	0.12
Social withdrawal	3	3	0.6
Decline in cognitive function	15	13	0.4
Possibility of depression	11	13	0.4
Pre-frail and frail	20	28	0.058

*POP-Q* Pelvic Organ Prolapse Quantification

**Fig. 2:**
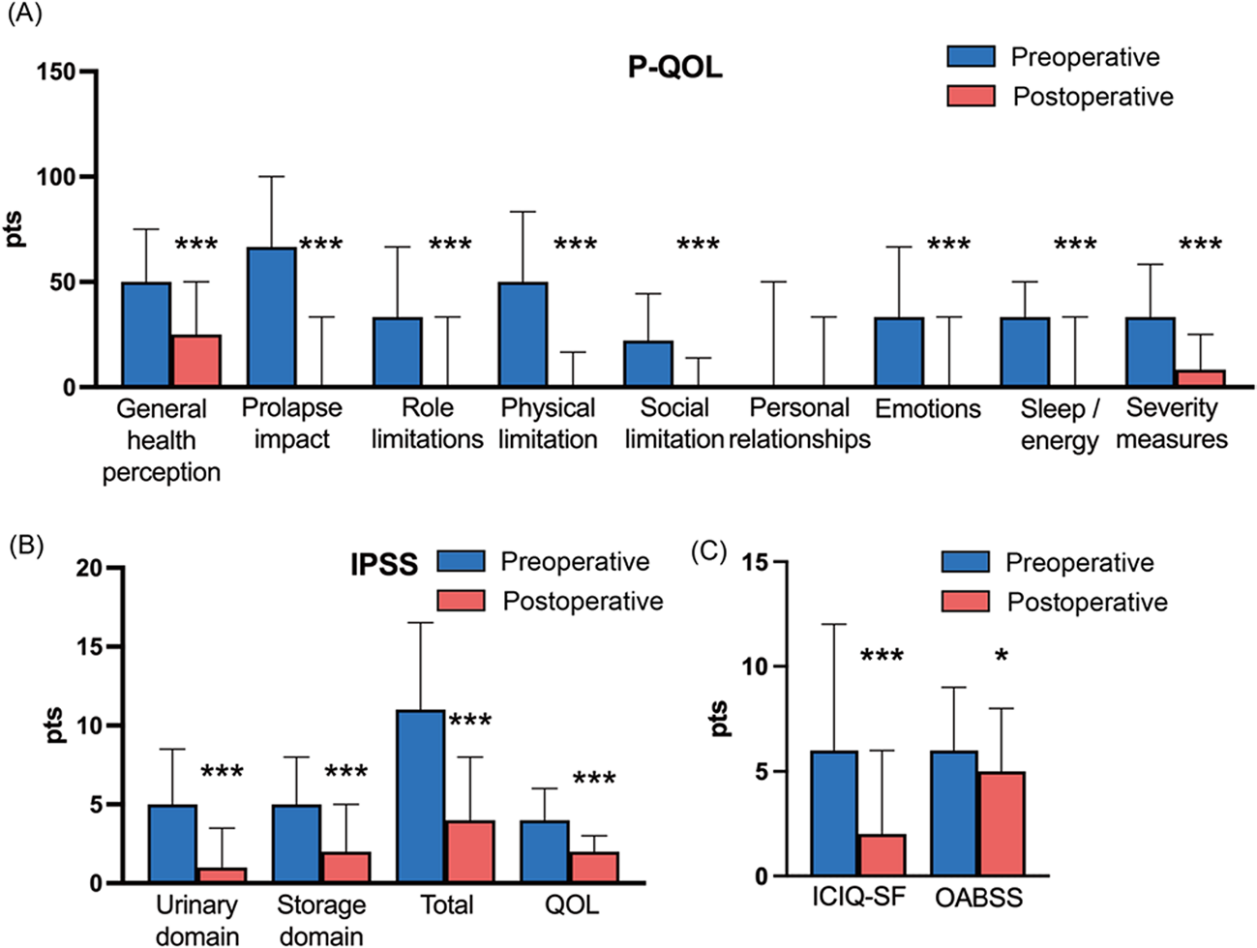
Pre- and postoperative comparisons. **A** Pelvic Organ Prolapse Quality of Life (P-QOL), **B** International Prostate Symptom Score (IPSS), **C** Incontinence Symptom Questionnaire (ICIQ-SF) and Overactive Bladder Symptom Score (OABSS). In each graph, the bar indicates median with interquartile range. *p < 0.05 and ***p < 0.001 are represented

Preoperative and postoperative evaluations of the 65 patients who underwent surgical treatment, including laparoscopic sacrocolpopexy (*n* = 56, 86.2%), colpocleisis (*n* = 2, 3.1%), tension-free vaginal tape (TVT; *n* = 4, 6.2%), and native tissue repair (*n* = 3, 4.6%), revealed no advanced postoperative POP-Q stage cases (Table 3). Although all cases revealed improvements in POP-Q stages, 13 patients remained at stage I or higher postoperatively. The objective success rate, defined as achieving a postoperative POP-Q stage of I or 0, was 58 out of 65 (89.2%). The number of cases exhibiting each condition listed on the Kihon Checklist declined postoperatively, except for social withdrawal; however, these changes did not reach statistical significance (Table 3). Furthermore, when postoperative frailty status was evaluated according to whether the patient had pre-frail or frail status preoperatively, a significantly greater number of patients showed an improvement in frailty status rather than a deterioration (Table 4; *p* < 0.001). Specifically, 15 patients (23.1%) showed improvement in frailty status, whereas only 3 patients (4.6%) experienced deterioration. Additionally, 30 patients (46.1%) maintained their nonfrail status postoperatively, and 17 (26.1%) showed no change in frailty status. Postoperatively, the P-QOL assessment revealed significant improvements in all domains, except for personal relationships (Fig. 2A; personal relationships: *p* = 0.12; all other domains: *p* < 0.001). The median (IQR) scores for P-QOL domains were as follows: general health perception (preoperative: 50 [25–75], postoperative: 25 [0–50]), prolapse impact (preoperative: 67 [33–100], postoperative: 0 [0–33]), role limitations (preoperative: 33 [0–67], postoperative: 0 [0–33]), physical limitations (preoperative: 50 [17–83], postoperative: 0 [0–17]), social limitations (preoperative: 22 [0–44], postoperative: 0 [0–14]), emotions (preoperative: 33 [11–67], postoperative: 0 [0–33]), sleep/energy (preoperative: 33 [8.4–50], postoperative: 0 [0–33]), and severity measures (preoperative: 33 [17–58], postoperative: 8.3 [0–25]). The IPSS demonstrated significant postoperative improvements in voiding, storage, total score, and QOL (Fig. 2B; all *p* < 0.001). The median (IQR) scores for IPSS were urinary domain (preoperative: 5 [1–8.5], postoperative: 1 [0–3.5]), storage domain (preoperative: 5 [3.5–8], postoperative: 2 [1–5]), total score (preoperative: 11 [5–17], postoperative: 4 [1.5–8]), and QOL (preoperative: 4 [3–6], postoperative: 2 [1–3]). Additionally, the ICIQ-SF and OABSS showed significant postoperative improvements (Fig. 2C; ICIQ-SF—preoperative: 6 [1–12], postoperative: 2 [1–6], *p* < 0.001; OABSS—preoperative: 6 [3–9], postoperative: 5 [0–8], *p* = 0.033).

**Table 3:** Comparison between pre- and postoperative changes (*n* = 65)

	Preoperative	Postoperative	*p* value
POP-Q stage			< 0.001
0	4	52	
I	0	6	
II	24	7	
III	28	0	
IV	9	0	
Kihon Checklist			
Decline in motor function	16	13	0.33
Malnutrition	0	0	–
Decline in oral function	8	7	0.5
Social withdrawal	4	6	0.37
Decline in cognitive function	16	15	0.5
Possibility of depression	18	10	0.067
Pre-frail and frail	34	24	0.077

*POP-Q* Pelvic Organ Prolapse Quantification

**Table 4:** Changes in frailty status between the pre- and postoperative period (*n* = 65)

	Preoperatively pre-frail and frail		
	Negative	Positive	*p* value
Changes in postoperative frailty status			
Deteriorated	1	2	< 0.001
No change	30	17	
Improved	–	15

**Fig. 3:**
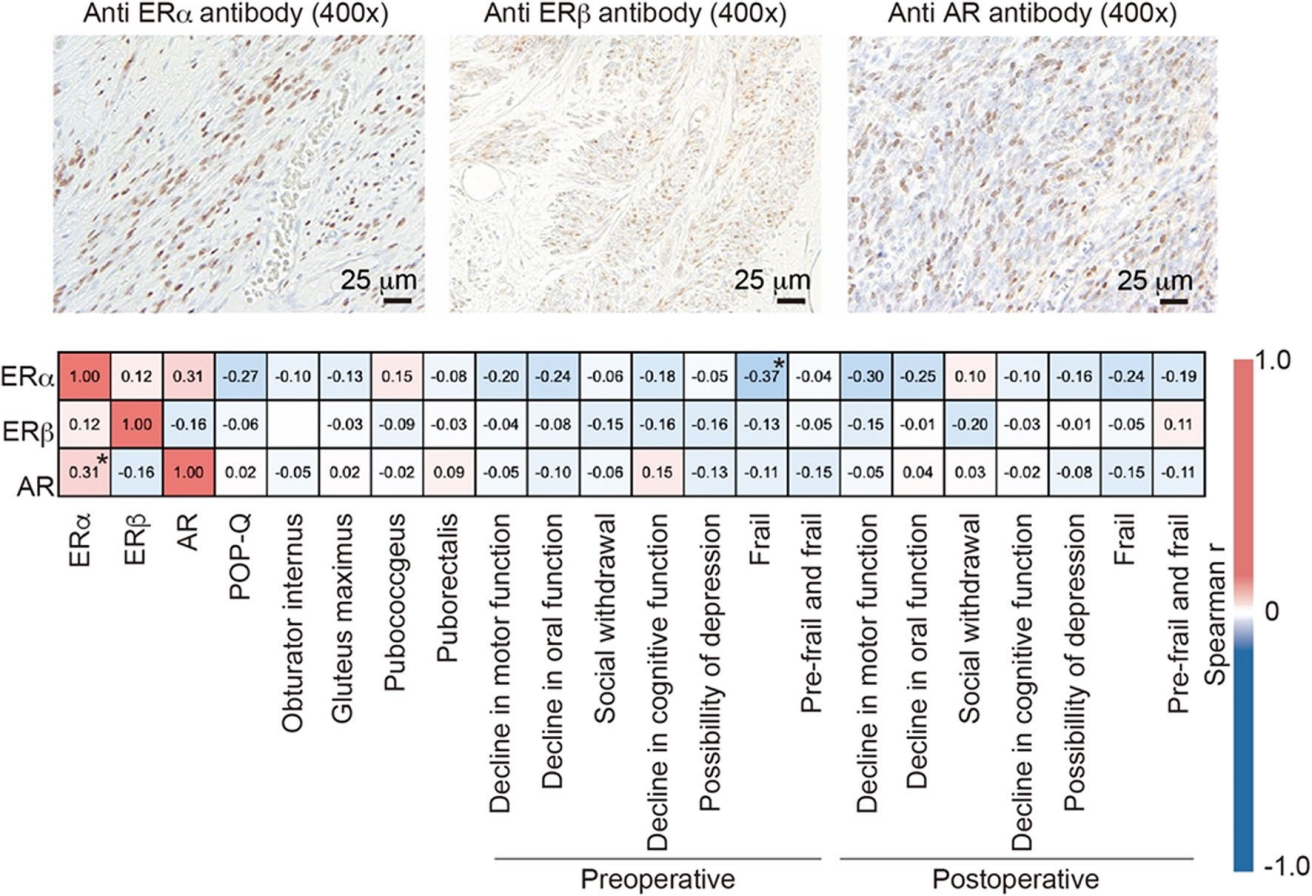
Correlation between sex hormone expression and each parameter. Sex hormone expression was examined using the immunoreactivity (IR) scores. Representative stained images and the degree of expression of each stain and the correlation coefficients are shown. *p < 0.05

In the 43 patients in whom immunostaining was performed on surgical specimens, there was a significant correlation between ERα and AR (Spearman *r* = 0.31, *p* = 0.04, Fig. 3), and between ER and the presence of preoperative frailty (Spearman *r* = −0.37, *p* = 0.014, Fig. 3). No statistically significant correlations were found between the various domains of the preoperative Kihon Checklist and ERα, ERβ, or AR levels (Fig. 3).

## Discussion

This study demonstrated that advanced POP was associated with frailty, muscle weakness, and poor QOL. Although no significant overall correlation was found between advanced POP and frailty, muscle weakness was significantly associated with advanced POP. Additionally, in a subset of patients who underwent surgery, advanced POP was significantly associated with higher rates of frailty. Our findings indicate that surgical interventions improve lower urinary tract dysfunction, QOL, and potentially reduce frailty in patients with POP. Although 46.2% of the patients maintained their nonfrail status and 23% showed improvement, 26.1% experienced no change, and 4.6% showed deterioration. Immunostaining linked ERα to preoperative frailty. These findings suggest that prolapse treatment may have a positive impact on frailty and overall well-being in some patients. Surgical intervention for POP not only addresses the mechanical issues but also contributes to reducing frailty. Additionally, the observed impact of lower sex hormone receptor levels on frailty suggests that hormone replacement therapy could be a potential avenue for mitigating frailty in postmenopausal women, warranting further investigation.

Although previous studies have indicated that advanced cases of POP present with worse IPSS and P-QOL [8–10], this study did not find a correlation between POP severity and IPSS. The relatively high IPSS values observed, even in cases of mild POP, may have influenced the discrepancy in this result. Additionally, the mean age in previous studies was 67 years, suggesting that age-related differences could affect urinary symptoms. In contrast, P-QOL was related to the POP stage, as in previous reports [8–10], suggesting that P-QOL may reflect a more comprehensive assessment of QOL. Similarly, ICIQ-SF and OABSS also correlated with the severity of illness, suggesting that the IPSS might not capture the overall impact of POP because of its age sensitivity and primary focus on voiding symptoms.

Muscle weakness on the Kihon Checklist was significantly associated with advanced POP. This suggests that POP might not only be a result of pelvic floor muscle weakening, but also of muscle weakening throughout the body. This is supported by previous research showing that frailty, sarcopenia, and functional disability are common in older women seeking treatment for pelvic floor dysfunction [27, 28]. Among the pelvic floor muscles that we focused on, the pubococcygeus showed a significant difference in its diameter, with patients with advanced POP having a significantly reduced pubococcygeus diameter compared with those with mild POP. The pubococcygeus, together with the iliococcygeus, is an important component of the pelvic floor muscle group. It contributes to the maintenance of urinary and defecatory functions and is known to be associated with POP [29, 30]. In addition, MRI observations of the obturator internus and gluteus maximus showed that they were not associated with POP severity despite being located in the same plane as the pelvic floor muscle group. Although these muscles are essential for lower extremity movement and are functionally related to the anorectal musculature [31, 32], it is likely that certain pelvic floor muscle groups, especially the anorectal musculature, play a more important role in POP severity. These results suggest that specific muscles within the pelvic floor musculature play a central role in the onset and progression of POP and that generalized muscle weakness may also be involved in the process leading to the severity of POP. Sarcopenia, frailty, POP, and pelvic floor muscle function are important themes in the health management of older adults. Although the diagnostic criteria for sarcopenia primarily focus on limb muscle strength [33], our study results suggest that pelvic floor muscle dysfunction and POP might play a significant role in the diagnosis of sarcopenia. Further research is needed, but the presence of POP could potentially serve as a simple indicator of sarcopenia and frailty. Addressing both localized and generalized muscle weakness could potentially improve patient outcomes and reduce the progression of POP. This highlights the need for a holistic approach to managing POP, incorporating both targeted pelvic floor muscle training and broader interventions to address overall muscle health.

Surgery for POP and urinary incontinence improved symptom scores regardless of severity. This is consistent with the results of previous studies [8–10] and suggests that older age might be closely related to overactive bladder symptoms. Additionally, ICIQ-SF scores improved postoperatively, possibly because of the inclusion of patients who underwent TVT surgery for urinary incontinence and the absence of POP. Interestingly, according to the Kihon Checklist, 15 of the 34 patients who had preoperative pre-frailty or frailty improved postoperatively. This suggests that the treatment of pelvic floor disorders, including incontinence and POP, might also be associated with improvements in conditions such as sarcopenia and frailty. In our cohort, the predominance of laparoscopic sacrocolpopexy reflected the specialized expertise of our facility and patient preferences, leading to a significant deviation from common practice in other regions. According to the 2020 total surgical numbers from the Japanese Ministry of Health, Labor, and Welfare, sacrocolpopexy significantly outnumbers vaginal wall repair (3,870 vs 985) [34]. Our facility places an emphasis on laparoscopic surgery, leading many patients to opt for laparoscopic sacrocolpopexy, which is chosen because of its lower recurrence rate and fewer complications. Native tissue repair is less frequently chosen because of concerns regarding higher recurrence rates. Additionally, sexual activity was not specifically assessed in this study, but our previous report using TVM cases highlighted extremely low sexual activity in Japanese patients with POP and elderly women [8]. This implies that sexual function was not a major determinant of the choice of surgical procedure in our cohort. Previous studies suggest that menopause might be an important risk factor for POP and that sex hormones play a major role in its etiology [35, 36]. Although associations among ERα, ERβ, and AR have been suggested previously, these associations have been inconsistent. The current study showed a nonsignificant but negative correlation between POP severity and sex hormone receptor levels. Consistent with the results of a previous report [13], this suggests that sex hormone receptors might play an inhibitory role in the progression of POP. Moreover, the present study showed that ERα has a particularly important influence on frailty. Therefore, ERα expression in the uterine adnexa may reflect systemic muscle function and may be an important indicator for preventing or improving frailty.

This study has several limitations. First, the sample size, though adequate for initial analyses, is relatively small and may not fully represent the broader population of elderly women with POP. The use of a retrospective mail survey for the collection of postoperative outcomes could introduce recall bias. Additionally, the immunohistochemical analysis was limited to those who provided specimens, which might not represent the entire surgical group, and did not take into consideration other factors that might influence hormone receptor expression. Moreover, the predominance of laparoscopic sacrocolpopexy in our cohort reflects the specific practice pattern of our facility, leading to potential bias in surgical outcomes. Consequently, this may limit the generalizability of our findings to other settings in which different surgical techniques, such as native tissue repair, are more commonly performed. Further research with a larger and more diverse sample, as well as more objective measures, is needed to confirm and expand on these findings.

## Conclusion

This study demonstrated a significant correlation between advanced POP and poor QOL, worse urinary symptoms, and motor function limitations. Postoperative improvements were significant in QOL and urinary symptoms. Additionally, ERα expression was linked to preoperative frailty. These findings suggest that treating POP can have a positive impact on patient outcomes and extend a healthy life.

## Supplementary Information

Below is the link to the electronic supplementary material.

Supplementary file1 (DOCX 15 KB)

## Data Availability

The data supporting the findings of this study are available from the corresponding author upon reasonable request.

## References

[1] Sullivan DF. A single index of mortality and morbidity. HSMHA Health Rep. 1971;86(4):347–54. 5554262 PMC1937122

[2] Cao X, Hou Y, Zhang X, Xu C, Jia P, Sun X, Sun L, Gao Y, Yang H, Cui Z, Wang Y, Wang Y. A comparative, correlate analysis and projection of global and regional life expectancy, healthy life expectancy, and their GAP: 1995–2025. J Glob Health. 2020;10(2):020407. 10.7189/jogh.10.020407. 33110572 10.7189/jogh.10.020407 PMC7568920

[3] Centers for Disease Control and Prevention. Trends in aging—United States and worldwide. MMWR Morb Mortal Wkly Rep. 2003;52(6):101–4, 106. 12645839

[4] Tokudome S, Hashimoto S, Igata A. Life expectancy and healthy life expectancy of Japan: the fastest graying society in the world. BMC Res Notes. 2016;9(1):482. 10.1186/s13104-016-2281-2. 27793196 10.1186/s13104-016-2281-2 PMC5084424

[5] Cooper C, Dere W, Evans W, Kanis JA, Rizzoli R, Sayer AA, Sieber CC, Kaufman JM, Abellan van Kan G, Boonen S, Adachi J, Mitlak B, Tsouderos Y, Rolland Y, Reginster JY. Frailty and sarcopenia: definitions and outcome parameters. Osteoporos Int. 2012;23(7):1839–48. 10.1007/s00198-012-1913-1. 22290243 10.1007/s00198-012-1913-1

[6] Nielsen CR, Ahrenfeldt LJ, Jeune B, Christensen K, Lindahl-Jacobsen R. Healthy life expectancy by frailty state in Europe from 2004 to 2015: findings from SHARE. Eur J Public Health. 2021;31(3):554–60. 10.1093/eurpub/ckab012. 33615329 10.1093/eurpub/ckab012 PMC8485734

[7] Shamliyan T, Talley KM, Ramakrishnan R, Kane RL. Association of frailty with survival: a systematic literature review. Ageing Res Rev. 2013;12(2):719–36. 10.1016/j.arr.2012.03.001. 22426304 10.1016/j.arr.2012.03.001

[8] Obinata D, Yamaguchi K, Hashimoto S, Yoshizawa T, Mochida J, Takahashi S. Tension-free vaginal mesh for patients with pelvic organ prolapse: mid-term functional outcomes. J Int Med Res. 2022;50(6):3000605221106434. 10.1177/03000605221106434. 35734995 10.1177/03000605221106434 PMC9235303

[9] Takahashi S, Obinata D, Sakuma T, Nagane Y, Sato K, Mochida J, Ichinose T, Yamaguchi K. Tension-free vaginal mesh procedure for pelvic organ prolapse: a single-center experience of 310 cases with 1-year follow up. Int J Urol. 2010;17(4):353–8. 10.1111/j.1442-2042.2010.02469.x. 20202001 10.1111/j.1442-2042.2010.02469.x

[10] Obinata D, Yamaguchi K, Ito A, Murata Y, Ashikari D, Igarashi T, Sato K, Mochida J, Yamanaka Y, Takahashi S. Lower urinary tract symptoms in female patients with pelvic organ prolapse: efficacy of pelvic floor reconstruction. Int J Urol. 2014;21(3):301–7. 10.1111/iju.12281. 24112546 10.1111/iju.12281

[11] Li C, Gong Y, Wang B. The efficacy of pelvic floor muscle training for pelvic organ prolapse: a systematic review and meta-analysis. Int Urogynecol J. 2016;27(7):981–92. 10.1007/s00192-015-2846-y. 26407564 10.1007/s00192-015-2846-y

[12] DeLancey JO, Morgan DM, Fenner DE, Kearney R, Guire K, Miller JM, Hussain H, Umek W, Hsu Y, Ashton-Miller JA. Comparison of levator ani muscle defects and function in women with and without pelvic organ prolapse. Obstet Gynecol. 2007;109(2 Pt 1):295–302. 10.1097/01.AOG.0000250901.57095.ba. 17267827 10.1097/01.AOG.0000250901.57095.ba

[13] Ikeda K, Horie-Inoue K, Inoue S. Functions of estrogen and estrogen receptor signaling on skeletal muscle. J Steroid Biochem Mol Biol. 2019;191:105375. 10.1016/j.jsbmb.2019.105375. 31067490 10.1016/j.jsbmb.2019.105375

[14] Bump RC, Mattiasson A, Bo K, Brubaker LP, DeLancey JO, Klarskov P, Shull BL, Smith AR. The standardization of terminology of female pelvic organ prolapse and pelvic floor dysfunction. Am J Obstet Gynecol. 1996;175(1):10–7. 8694033 10.1016/s0002-9378(96)70243-0

[15] Iwai-Saito K, Sato K, Aida J, Kondo K. Association of frailty with influenza and hospitalization due to influenza among independent older adults: a longitudinal study of Japan Gerontological Evaluation Study (JAGES). BMC Geriatr. 2023;23(1):249. 10.1186/s12877-023-03979-y. 37101153 10.1186/s12877-023-03979-y PMC10131426

[16] Satake S, Senda K, Hong YJ, Miura H, Endo H, Sakurai T, Kondo I, Toba K. Validity of the Kihon checklist for assessing frailty status. Geriatr Gerontol Int. 2016;16(6):709–15. 10.1111/ggi.12543. 26171645 10.1111/ggi.12543

[17] Digesu GA, Khullar V, Cardozo L, Robinson D, Salvatore S. P-QOL: a validated questionnaire to assess the symptoms and quality of life of women with urogenital prolapse. Int Urogynecol J Pelvic Floor Dysfunct. 2005;16(3):176–81; discussion 181. 10.1007/s00192-004-1225-x. 15875234 10.1007/s00192-004-1225-x

[18] Kinjo M, Yoshimura Y, Kitagawa Y, Okegawa T, Nutahara K. Sexual activity and quality of life in Japanese pelvic organ prolapse patients after transvaginal mesh surgery. J Obstet Gynaecol Res. 2018;44(7):1302–7. 10.1111/jog.13654. 29672997 10.1111/jog.13654

[19] Barry MJ, Fowler FJ Jr, O'Leary MP, Bruskewitz RC, Holtgrewe HL, Mebust WK, Cockett AT. The American Urological Association symptom index for benign prostatic hyperplasia. The measurement committee of the American Urological Association. J Urol. 1992;148(5):1549–57; discussion 1564. 10.1016/s0022-5347(17)36966-5. 1279218 10.1016/s0022-5347(17)36966-5

[20] Homma Y, Yoshida M, Seki N, Yokoyama O, Kakizaki H, Gotoh M, Yamanishi T, Yamaguchi O, Takeda M, Nishizawa O. Symptom assessment tool for overactive bladder syndrome–overactive bladder symptom score. Urology. 2006;68(2):318–23. 10.1016/j.urology.2006.02.042. 16904444 10.1016/j.urology.2006.02.042

[21] Karantanis E, Fynes M, Moore KH, Stanton SL. Comparison of the ICIQ-SF and 24-hour pad test with other measures for evaluating the severity of urodynamic stress incontinence. Int Urogynecol J Pelvic Floor Dysfunct. 2004;15(2):111–6; discussion 116. 10.1007/s00192-004-1123-2. 15014938 10.1007/s00192-004-1123-2

[22] Caagbay D, Fatakia FT, Dietz HP, Raynes-Greenow C, Martinho N, Black KI. Is pelvic floor muscle strength and thickness associated with pelvic organ prolapse in Nepali women?—A cross-sectional study. Braz J Phys Ther. 2021;25(2):214–20. 10.1016/j.bjpt.2020.05.011. 32563664 10.1016/j.bjpt.2020.05.011 PMC7990730

[23] Obinata D, Takayama K, Urano T, Murata T, Kumagai J, Fujimura T, Ikeda K, Horie-Inoue K, Homma Y, Ouchi Y, Takahashi S, Inoue S. Oct1 regulates cell growth of LNCaP cells and is a prognostic factor for prostate cancer. Int J Cancer. 2012;130(5):1021–8. 10.1002/ijc.26043. 21387309 10.1002/ijc.26043

[24] Obinata D, Takada S, Takayama K, Urano T, Ito A, Ashikari D, Fujiwara K, Yamada Y, Murata T, Kumagai J, Fujimura T, Ikeda K, Horie-Inoue K, Homma Y, Takahashi S, Inoue S. Abhydrolase domain containing 2, an androgen target gene, promotes prostate cancer cell proliferation and migration. Eur J Cancer. 2016;57:39–49. 10.1016/j.ejca.2016.01.002. 26854828 10.1016/j.ejca.2016.01.002

[25] Aydin YM, Sahin AB, Dolek R, Vuruskan BA, Ocakoglu G, Vuruskan H, Yavascaoglu I, Coskun B. Prognostic value of estrogen receptors in patients who underwent prostatectomy for non-metastatic prostate cancer. Oncol Lett. 2023;25(2):78. 10.3892/ol.2023.13664. 36742361 10.3892/ol.2023.13664 PMC9853097

[26] Faul F, Erdfelder E, Lang AG, Buchner A. G*Power 3: a flexible statistical power analysis program for the social, behavioral, and biomedical sciences. Behav Res Methods. 2007;39(2):175–91. 10.3758/bf03193146. 17695343 10.3758/bf03193146

[27] Erekson EA, Fried TR, Martin DK, Rutherford TJ, Strohbehn K, Bynum JP. Frailty, cognitive impairment, and functional disability in older women with female pelvic floor dysfunction. Int Urogynecol J. 2015;26(6):823–30. 10.1007/s00192-014-2596-2. 25516232 10.1007/s00192-014-2596-2 PMC4713028

[28] Silva RRL, Coutinho JFV, Vasconcelos CTM, Vasconcelos Neto JA, Barbosa RGB, Marques MB, Saboia DM, Maia JC. Prevalence of sarcopenia in older women with pelvic floor dysfunction. Eur J Obstet Gynecol Reprod Biol. 2021;263:159–63. 10.1016/j.ejogrb.2021.06.037. 34218202 10.1016/j.ejogrb.2021.06.037

[29] Visco AG, Yuan L. Differential gene expression in pubococcygeus muscle from patients with pelvic organ prolapse. Am J Obstet Gynecol. 2003;189(1):102–12. 10.1067/mob.2003.372. 12861146 10.1067/mob.2003.372

[30] Branham V, Thomas J, Jaffe T, Crockett M, South M, Jamison M, Weidner A. Levator ani abnormality 6 weeks after delivery persists at 6 months. Am J Obstet Gynecol. 2007;197(1):65.e1–6. 10.1016/j.ajog.2007.02.040. 17618761 10.1016/j.ajog.2007.02.040 PMC2601553

[31] Soljanik I, Janssen U, May F, Fritsch H, Stief CG, Weissenbacher ER, Friese K, Lienemann A. Functional interactions between the fossa ischioanalis, levator ani and gluteus maximus muscles of the female pelvic floor: a prospective study in nulliparous women. Arch Gynecol Obstet. 2012;286(4):931–8. 10.1007/s00404-012-2377-4. 22692630 10.1007/s00404-012-2377-4

[32] Tuttle LJ, DeLozier ER, Harter KA, Johnson SA, Plotts CN, Swartz JL. The role of the obturator internus muscle in pelvic floor function. J Womens Health Phys Ther. 2016;40(1):15–9.

[33] Davalos-Yerovi V, Marco E, Sanchez-Rodriguez D, Guillen-Sola A, Duran X, Pascual EM, Muniesa JM, Escalada F, Duarte E. Sarcopenia according to the revised European consensus on definition and diagnosis (EWGSOP2) criteria predicts hospitalizations and long-term mortality in rehabilitation patients with stable chronic obstructive pulmonary disease. J Am Med Dir Assoc. 2019;20(8):1047–9. 10.1016/j.jamda.2019.03.019. 31133471 10.1016/j.jamda.2019.03.019

[34] Mukai R, Shimada K, Suzuki T, Nakao S, Tanaka M, Matsumoto K, Yoshida Y, Goto F, Inoue M, Satake R, Nishibata Y, Sugihara H, Nakamura M. Trends associated with hemorrhoids in Japan: data mining of medical information datasets and the national database of health insurance claims and specific health checkups of Japan (NDB) Open Data Japan. Biol Pharm Bull. 2020;43(12):1831–8. 10.1248/bpb.b20-00157. 33268700 10.1248/bpb.b20-00157

[35] Soderberg MW, Johansson B, Masironi B, Bystrom B, Falconer C, Sahlin L, Ordeberg GE. Pelvic floor sex steroid hormone receptors, distribution and expression in pre- and postmenopausal stress urinary incontinent women. Acta Obstet Gynecol Scand. 2007;86(11):1377–84. 10.1080/00016340701625446. 17963065 10.1080/00016340701625446

[36] Ward RM, Velez Edwards DR, Edwards T, Giri A, Jerome RN, Wu JM. Genetic epidemiology of pelvic organ prolapse: a systematic review. Am J Obstet Gynecol. 2014;211(4):326–35. 10.1016/j.ajog.2014.04.006. 24721264 10.1016/j.ajog.2014.04.006 PMC4213176

