# Supplementary material for: Association Between Frailty and Pelvic Organ Prolapse in Elderly Women: A Retrospective Study
Source: Int Urogynecol J. 2024 Aug 26;35(9):1889–98. doi: 10.1007/s00192-024-05898-x (PMC11420369; doi:10.1007/s00192-024-05898-x)
Supplement: Supplementary file 1 — Supplementary file1 (DOCX 15 KB) [file 192_2024_5898_MOESM1_ESM.docx]

| Supplementary Table 1. Comparison of the Kihon Checklist results between mild and severe surgical cases (n = 65) | | | |
| --- | --- | --- | --- |
|  | POP-Q | |  |
| Kihon check list | Mild  Stage ≤ II  n=28 | Advanced  Stage ≥ III  n=37 | p value |
| Decline in motor function | 2 | 14 | 0.004 |
| Malnutrition | 0 | 0 | - |
| Decline in oral function | 1 | 7 | 0.065 |
| Social withdrawal | 1 | 3 | 0.4 |
| Decline in cognitive function | 5 | 11 | 0.21 |
| Possibility of depression | 7 | 11 | 0.4 |
| Pre-frail and frail | 10 | 24 | 0.018 |
